# Health literacy and health-related quality of life: The mediating role of irrational happiness

**DOI:** 10.1515/med-2025-1148

**Published:** 2025-02-12

**Authors:** Amil Huseynov, Begum Satici

**Affiliations:** Department of Transplantation, Istanbul Medicana International, Istanbul, Türkiye; Department of Psychological Counseling, Yildiz Technical University, Istanbul, Türkiye

**Keywords:** health-related quality of life, health literacy, irrational happiness, structural equation modeling

## Abstract

**Background:** Health literacy (HL) and irrational beliefs about happiness significantly influence adults’ health-related quality of life (HRQoL). The purpose of this study was to examine whether irrational happiness mediates the relationship between HL and HRQoL among adults.

**Methods:** A total of 686 adults (468 women and 218 men; mean age = 22.30  ±  6.83 years) completed self-report questionnaires, including the Health Literacy Scale-Short Form, the EUROHIS-QOL 8, and the Irrational Happiness Beliefs Scale. Data were analyzed using structural equation modeling and bootstrapping methods.

**Results:** HL had both direct (*β* = 0.260, *p* < 0.01) and indirect effects on HRQoL. HL directly predicted irrational happiness (*β* = –0.369, *p* < 0.01), which in turn directly predicted HRQoL (*β* = –0.318, *p* < 0.01). Specifically, irrational happiness significantly mediated the relationship between HL and HRQoL (bootstrap coefficient = 0.117, 95% CI = 0.071–0.175).

**Conclusion:** These findings suggest that interventions aiming to enhance adults’ HRQoL should consider not only improving HL but also addressing irrational beliefs about happiness.

## 1 Introduction

Health-related quality of life (HRQoL) refers to individuals’ subjective evaluations of how their physical, mental, emotional, and social health statuses affect their daily lives [1]. HRQoL is not confined merely to the presence or absence of medical conditions; it also encompasses how well individuals feel, their functionality, and their social participation [2]. In modern healthcare, the significance of HRQoL has increasingly been recognized, and it has become a widespread indicator for assessing the success of health interventions [3]. Psychological and social health emerge as determining factors of HRQoL, just as much as physical health does [4]. Research indicates that individuals with higher HRQoL not only tend to live longer but also exhibit greater satisfaction and happiness in their daily lives [5,6,7]. Therefore, HRQoL is an essential health indicator that allows for a more holistic consideration of individuals’ health statuses. A good HRQoL is critically important for the management of chronic diseases, healthy aging, and the enhancement of overall life satisfaction [8,9].

Health literacy (HL) refers to individuals’ abilities to acquire, understand, and use health-related information to make appropriate decisions [10]. Individuals with high HL participate more effectively in health systems and can better manage their own health conditions [11,12]. Studies have shown that HL improves individuals’ disease prevention behaviors, facilitates coping with chronic conditions, and enhances overall quality of life [12,13,14]. Particularly in situations where HL is low, individuals are more likely to misinterpret important health information, which can negatively impact health outcomes [15].

HL not only contributes to individuals’ physical and mental health but also plays a significant role in enhancing their HRQoL [16]. In this context, it has been observed that individuals with high HL not only experience better quality of life but also exhibit increased trust in healthcare services [17]. To better understand the relationship between HL and HRQoL, it is necessary to examine individuals’ emotional and cognitive processes. This is where the concept of irrational happiness comes into play, serving as a potential mediator that shapes the connection between the knowledge gained from HL and the quality of life.

### 1.1 The mediating role of irrational happiness

Irrational happiness is defined as the display of unrealistic optimism by individuals despite adverse health conditions or life challenges [18]. However, this type of happiness is considered a maladaptive trait rather than an adaptive one [19]; it may lead individuals to not take health-related threats seriously enough and to postpone appropriate health behaviors. While irrational happiness can enhance emotional comfort, such excessive optimism increases the likelihood of underestimating one’s health status and engaging in risky behaviors.

In this study, irrational happiness was found to function as a negative mediating mechanism in the relationship between HL and HRQoL. High HL enables individuals to assess health risks more accurately, whereas those with high levels of irrational happiness may tend to ignore or misinterpret this information. For example, excessive optimism might lead them to disregard health warnings and miss opportunities for early intervention [20]. Therefore, irrational happiness can prevent individuals from fully benefiting from their levels of HL, potentially creating a negative impact on HRQoL.

### 1.2 The present study

This study aims to examine the relationship between HL and HRQoL, and to investigate the mediating role of irrational happiness in this relationship. The literature has demonstrated that HL directly affects individuals’ health behaviors and health outcomes [11,12,14]. However, studies are limited regarding the role psychological factors, especially irrational happiness, play in the process by which individuals’ health knowledge translates into HRQoL. This research takes an important step toward deeply understanding the impact of irrational happiness on individuals’ quality of life. In line with this, we have developed the following hypotheses in our study:

**Hypothesis 1:** HL has a positive impact on HRQoL.

**Hypothesis 2:** Irrational happiness has a direct impact on HRQoL.

**Hypothesis 3:** Irrational happiness mediates the relationship between HL and HRQoL.

## 2 Method

### 2.1 Participants

A total of 686 individuals aged between 18 and 56 participated in this study (mean age = 22.30, SD = 6.83). The sample consisted of 468 females (68.2%) and 218 males (31.8%). Based on socioeconomic status (SES), 73 participants (10.6%) were of low SES, 573 (83.5%) were of middle SES, and 40 (5.8%) were of high SES. In terms of educational attainment, 17 participants (2.5%) had completed high school, 627 participants (91.4%) held a bachelor’s degree, 27 participants (3.9%) had a master’s degree, and 15 participants (2.2%) held a doctoral degree. Data were collected in August and September 2024 from five different cities in Türkiye, representing small, medium, and large urban areas, using a convenience sampling method. The study was conducted in a digital environment. Approximately 56% of the participants reported having a chronic illness, while the remaining participants did not have an active illness at the time of the study. Regarding chronic health issues in the family, 411 participants (59.9%) reported having a family member with a chronic health problem, while 275 (40.1%) reported none. In the past 6 months, 87.5% of participants had visited a hospital at least once, and 20% had visited four times or more.

### 2.2 Measures

The Health Literacy Scale-Short Form (HLS-SF) was developed by Duong et al. [21] to measure individuals’ abilities to acquire, understand, evaluate, and apply health-related information. The Turkish adaptation and validity–reliability study of the scale have made it applicable to Turkish-speaking populations [22]. Studies have found the scale’s internal consistency reliability coefficient (Cronbach’s *α*) to be 0.856, and factor analyses have supported its structural validity [22].

The EUROHIS (WHOQOL-8.Tr) is an 8-item index measuring quality of life, constructed by selecting specific items from the World Health Organization Quality of Life Scale (WHOQOL). This scale aims to quickly and effectively assess individuals’ general quality of life and health status [23]. Studies on the psychometric properties of the Turkish version have demonstrated a high level of reliability, with an internal consistency reliability coefficient (Cronbach’s *α*) of 0.85. Structural validity analyses also support that the scale is a valid and reliable measurement tool within the Turkish population [23].

The Irrational Happiness Beliefs Scale (IHB) was developed to assess individuals’ irrational beliefs about happiness and their impact on subjective well-being [24]. Exploratory and confirmatory factor analyses confirmed that the IHB is unidimensional, consisting of three items with a high internal consistency (Cronbach’s *α* = 0.84). The scale showed significant positive correlations with measures of valuing happiness, negative affect, perceived stress, and irrational thinking. Conversely, it had significant negative correlations with satisfaction with life, subjective happiness, positive affect, psychological well-being, and rational thinking [24].

### 2.3 Data analysis

Initially, correlation analysis and descriptive statistics were conducted, followed by structural equation modeling (SEM). Adopting Kline’s [25] recommendations, a two-step SEM approach was employed. In the first step, we tested the measurement model to confirm whether the observed indicators effectively formed the latent variables and whether the relationships among these latent variables were valid. After validating the measurement model, we proceeded to test the hypothesized structural model. All analyses were performed using IBM SPSS 22 for descriptive statistics and correlation analyses and AMOS Graphics for SEM. Prior to SEM, the normality of the data was assessed via skewness and kurtosis values, which fell within acceptable ranges.

To evaluate the SEM results, we considered the goodness-of-fit indices recommended by Hu and Bentler [26], including chi-square (*χ*²) and degrees of freedom, as well as comparative fit index (CFI), normed fit index (NFI), Tucker-Lewis index (TLI), standardized root mean square residual (SRMR), root mean square error of approximation (RMSEA) values. The critical thresholds were set as follows: a *χ*²/d*f* ratio less than 5; CFI, NFI, and TLI values greater than 0.90; and SRMR and RMSEA values less than 0.80 [26]

In addition to SEM, we employed the increasingly popular bootstrapping procedure to provide additional evidence for the significance of mediation. Using bootstrapping with 10,000 resamples, we generated bootstrap estimates and confidence intervals (CIs). The absence of zero within these CIs indicates that the tested mediation effect is significant. All measurement scales demonstrated satisfactory reliability (Cronbach’s *α* > 0.70), further supporting the robustness of our analyses.


**Ethical approval:** The study was conducted in accordance with the principles of the Helsinki Declaration, and informed consent was obtained from all participants. Ethical approval for this research was obtained from the Yıldız Technical University Ethics Committee (ID = 2024-10/0335).

## 3 Results

Table 1 presents the descriptive statistics and Pearson correlations for the variables examined: HL, HRQoL, and irrational happiness. The Pearson correlation analysis revealed several significant relationships among the variables. HL was positively correlated with HRQoL (*r* = 0.329, *p* < 0.001), indicating that individuals with higher HL tend to report better quality of life related to their health. In contrast, HL was negatively associated with irrational happiness (*r* = −0.309, *p* < 0.001), suggesting that higher HL is linked to lower levels of irrational happiness. Additionally, HRQoL was negatively correlated with irrational happiness (*r* = −0.358, *p* < 0.001).

**Table 1:** Relationships among variables and descriptive statistics

Variables	1	2	3
1. HL	—		
2. HRQoL	0.329^**^	—	
3. Irrational happiness	−0.309^**^	−0.358^**^	—
Mean	34.62	27.27	9.74
SD	4.79	4.43	2.11
Skewness	−0.229	−0.530	0.865
Kurtosis	1.68	0.892	1.48
Cronbach *α*	0.789	0.816	0.702

^**^ *p* < 0.001.

### 3.1 Measurement model

The measurement model consisted of three latent variables – HL, HRQoL, and irrational happiness – with a total of seven observed variables: two each for HL and HRQoL, and three for irrational happiness. The results indicated a good fit for the measurement model, *χ*²(11, *N* = 686) = 21.77, *p* < 0.001, *χ*²/df = 1.98; GFI = 0.991; NFI = 0.987; CFI = 0.994; TLI = 0.988; RFI = 0.975; TLI = 0.988; SRMR = 0.025; RMSEA = 0.038. Additionally, factor loadings ranged from 0.525 to 0.918, suggesting that the observed variables significantly represented the latent constructs.

### 3.2 Structural model

**Figure 1:**
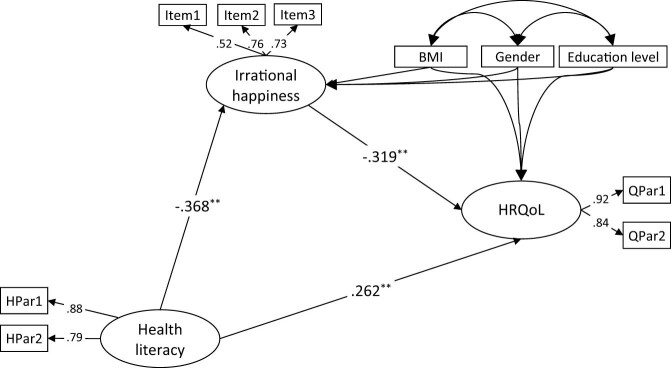
Standardized factor loadings for the structural model. Note. *N* = 686; ^**^ *p* < 0.01.

In the structural model, we tested whether irrational happiness mediates the relationship between HL and HRQoL, including BMI, gender, and education level as control variables. The model with irrational happiness as a mediator demonstrated a good fit: *χ*²(26, *N* = 686) = 56.82, *p* < 0.001; *χ*²/df = 2.18; GFI = 0.984; NFI = 0.968; CFI = 0.982; TLI = 0.969; RFI = 0.945; SRMR = 0.034; RMSEA = 0.042. Results indicated that HL directly and positively predicted HRQoL (*β* = 0.262, *p* < 0.01). Conversely, HL directly and negatively predicted irrational happiness (*β* = −0.368, *p* < 0.01), which in turn directly and negatively predicted HRQoL (*β* = −0.319, *p* < 0.01). The bootstrapping analysis revealed that the indirect path coefficient was significant (bootstrap coefficient = 0.117, 95% CI = 0.072–0.172). Taking all these findings into account, it can be concluded that irrational happiness mediates the relationship between HL and HRQoL. The path coefficients for this model are presented in Figure 1.

## 4 Discussion

This study investigated whether irrational happiness mediates the relationship between HL and HRQoL. Previous research aimed to identify and enhance the predictors of HRQoL when these variables were not considered together. According to the results of the study, the formulated hypotheses were confirmed and supported by structural equation modeling. The findings are discussed in detail below.

The first finding of the study indicates that HL has a positive effect on HRQoL. This result is consistent with findings in the literature that demonstrate a strong relationship between HL and individuals’ ability to manage their health conditions more effectively [10,17]. Individuals with high HL improve their treatment adherence and participate more actively in the healthcare system due to their capacity to understand and apply medical information [11,14]. This, in turn, enhances their physical and mental health levels, thereby increasing their HRQoL [3]. Research shows that individuals with high HL perform better not only in disease prevention and management processes but also in areas such as social participation and overall life satisfaction [12,13]. Consequently, these individuals can benefit more from the healthcare system by acting more consciously when accessing health services, leading to an improvement in their HRQoL.

In our study, Hypothesis 2, which proposed that irrational happiness has a direct negative effect on HRQoL, was confirmed. This finding indicates that irrational happiness does not always yield positive outcomes and that unrealistic optimism regarding individuals’ health conditions can produce negative effects. Specifically, irrational happiness is thought to increase the risk of underestimating the seriousness of health conditions and delaying necessary medical interventions. This finding aligns with previous research suggesting that emotions do not always facilitate making healthy decisions. For example, states of excessive optimism and irrational happiness can lead individuals to downplay their health problems, postpone seeking treatment, or ignore health-related risks [20]. Although irrational happiness may provide short-term emotional comfort, it can function as a cognitive bias that reduces HRQoL in the long term.

The findings support Hypothesis 3, demonstrating that irrational happiness mediates the relationship between HL and HRQoL. This result reveals that individuals with high HL experience lower levels of irrational happiness, which helps them make more balanced health decisions. High HL increases individuals’ access to and understanding of information about their health conditions, thereby reducing the use of defensive psychological mechanisms such as irrational happiness. This situation supports the notion that a lack of health knowledge can increase unrealistic optimism [27] and sustainably reduce individuals’ HRQoL [13,14,15]. In other words, a decrease in irrational happiness may lead individuals to misassess their health conditions and develop inappropriate health behaviors. Therefore, it can be stated that among individuals with high HL, irrational happiness may decrease, and by making more conscious and balanced choices in health decisions, they can positively influence their HRQoL levels.

## 5 Implications

This study has demonstrated that HL not only encompasses the capacity to acquire health-related information but also holds the potential to enhance individuals’ quality of life. The findings suggest that HL can reduce irrational happiness, thereby improving HRQoL. This is particularly significant for public health policies and health education programs. It is recommended that educational campaigns should not be limited to merely conveying information but should also consider psychological tendencies like irrational happiness to provide individuals with more realistic health perspectives. Additionally, health professionals and policymakers can offer guidance aimed at balancing individuals’ psychological well-being while providing accessible information platforms to improve HL. This approach will help patients develop an evidence-based yet realistic and sustainable quality of life strategy to improve long-term health outcomes.

## 6 Conclusion

This study has elucidated the relationship between HL and HRQoL, highlighting the mediating role of irrational happiness in this relationship. The findings indicate that HL directly enhances quality of life by increasing individuals’ access to health-related information and improving their decision-making abilities. At the same time, it has been observed that irrational happiness can lead to excessive optimism and underestimation of health conditions, which may negatively impact HRQoL. Consequently, enhancing HL is a critical tool to support individuals in making health decisions that are both realistic and balanced.
